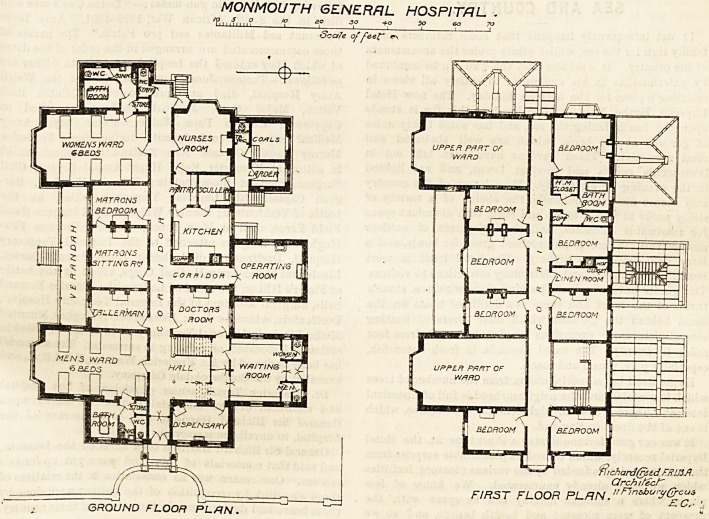# The New Hospital at Monmouth

**Published:** 1903-07-11

**Authors:** 


### HOSPITAL ADMINISTRATION.

### CONSTRUCTION AND ECONOMICS.

### THE NEW HOSPITAL AT MONMOUTH.

THIS building consists of one block, of which the main elevation faces south. It is divided from east to west by a corridor with an entrance at each end, but that on the east is the main one. The corridor gets some light from the staircase windows and from a window at its west end.

Beginning at the east end of the corridor there is a section of the building on the left, on entering, which contains bath-room, closet, sink, and a small store-room. This section ought to have been a block by itself and connected with the main by a short cross-ventilated passage, thus cutting it off effectually from the wards. Next is a ward for six beds. It has a good-sized bay window to the south and a small window on each side, one of which looks into the verandah. More than half of the ward is jammed between the bath-room section, the corridor wall, and a room marked "Tallerman" on the plan. Hence that end of the ward has no cross ventilation at all and cannot fail to be unsuitable for its purpose, as four of the beds are placed against dead walls. It is quite behind the age and sets modern hospital construction at defiance. No ward can be described as correct in principle unless it have a window on each side of each bed.

Further on are the matron's rooms, with the verandah in front, and then another ward for six beds, with the same faults as its fellow. The sanitary section at the west end has also the same defects as those already noticed. On the north side of the corridor going from the east are the dispensary, the hall, the waiting-room, the doctor's room, a corridor leading to the operating-room, the kitchen, pantry and nurses' room. All these may pass muster. The operating-room is properly placed to the north, and is well lighted by three windows and a roof light. Its walls are covered with glazed tiles, and the angles of the room are rounded off. So far as can be judged from the plan, it is the best part of the hospital. There is, however, no anæsthetic room, but in a small hospital this may not be much missed.

The nurses' duty-room is at the opposite side of the corridor from the women's ward, instead of being placed so that it could overlook the ward, and there is no nurses' room at all attached to the men's ward.

We look upon the defects we have pointed out as grave ones. They are in fact inexcusable in so small a hospital and where there can be no question of a restricted site.

The first floor contains eight bedrooms, bath-room, etc. As all these bedrooms can hardly be needed for the staff, it is intended that two may be used as wards when required. The store-room accommodation on both floors seems to be very small, quite inadequate in fact.

The floors of the wards are of fireproof construction, and are covered with pitch-pine blocks, while the corridors are laid with terrazzo. The walls are finished with cement plaster, and have the angles rounded off.

The warming is very properly by open fireplaces assisted when necessary by hot-water radiators. The wards have ventilating fresh air inlets on the floor level, and ventilating foul air outlets under the ceiling; but we are not told on whose system these are constructed, nor by what means they will be made to serve their respective purposes. The electric light is used.

The elevations are covered with rough cast on the lower story, and with Kentish tiles on the upper story. The drainage and sanitary fittings include all the latest improvements,

The architect is Mr. Richard Creed of Finsbury Circus.

## Figures and Tables

**Figure f1:**